# Improving Sensitivity and Longevity of In Vivo Glutamate Sensors with Electrodeposited NanoPt

**DOI:** 10.1021/acsami.4c06692

**Published:** 2024-07-30

**Authors:** Elaine M. Robbins, Benjamin Wong, May Yoon Pwint, Siamak Salavatian, Aman Mahajan, Xinyan Tracy Cui

**Affiliations:** † Department of Bioengineering, University of Pittsburgh, Pittsburgh, Pennsylvania 15261, United States; ‡ Department of Anesthesiology & Perioperative Medicine, University of Pittsburgh School of Medicine, Pittsburgh, Pennsylvania 15261, United States; § Center for Neural Basis of Cognition, University of Pittsburgh, Pittsburgh, Pennsylvania 15261, United States; ∥ McGowan Institute for Regenerative Medicine, University of Pittsburgh, Pittsburgh, Pennsylvania 15261, United States

**Keywords:** glutamate sensor, in vivo sensing, enzymatic sensor stability, nanoplatinum, GABA sensor, surface modification

## Abstract

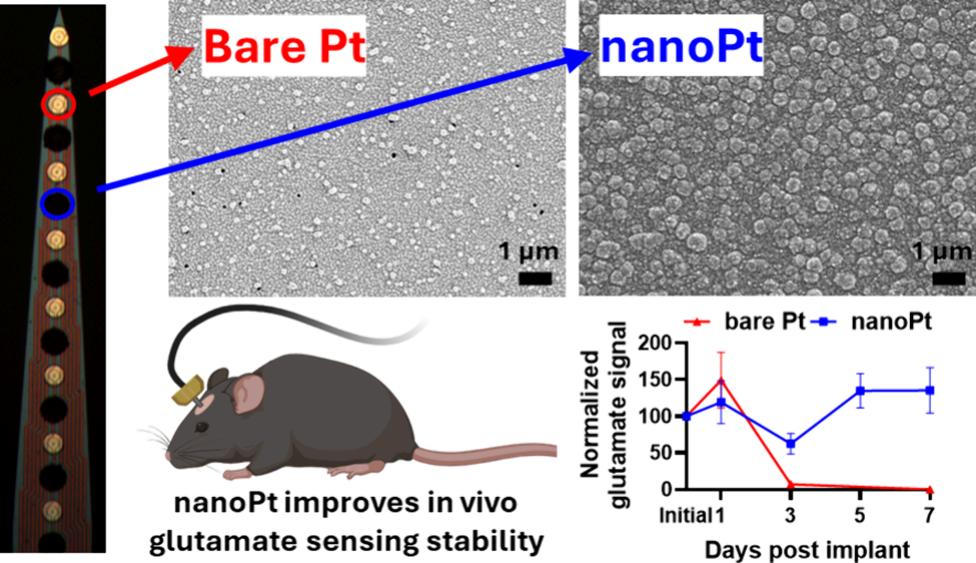

In vivo glutamate sensing has provided valuable insight into the physiology and pathology of the brain. Electrochemical glutamate biosensors, constructed by cross-linking glutamate oxidase onto an electrode and oxidizing H_2_O_2_ as a proxy for glutamate, are the gold standard for in vivo glutamate measurements for many applications. While glutamate sensors have been employed ubiquitously for acute measurements, there are almost no reports of long-term, chronic glutamate sensing in vivo, despite demonstrations of glutamate sensors lasting for weeks in vitro. To address this, we utilized a platinum electrode with nanometer-scale roughness (nanoPt) to improve the glutamate sensors’ sensitivity and longevity. NanoPt improved the GLU sensitivity by 67.4% and the sensors were stable in vitro for 3 weeks. In vivo, nanoPt glutamate sensors had a measurable signal above a control electrode on the same array for 7 days. We demonstrate the utility of the nanoPt sensors by studying the effect of traumatic brain injury on glutamate in the rat striatum with a flexible electrode array and report measurements of glutamate taken during the injury itself. We also show the flexibility of the nanoPt platform to be applied to other oxidase enzyme-based biosensors by measuring γ-aminobutyric acid in the porcine spinal cord. NanoPt is a simple, effective way to build high sensitivity, robust biosensors harnessing enzymes to detect neurotransmitters in vivo.

## Introduction

Glutamate (GLU) is an important neurotransmitter in the central nervous system. As the primary excitatory neurotransmitter in the brain, GLU is responsible for propagating diverse signals, including those related to pain^1^ and vision.^2^ Abnormal GLU signaling is associated with disease states including attention deficit hyperactive disorder,^3^ seizure,^4−6^ and excitotoxicity.^7−9^

In vivo GLU concentrations can be measured several ways. Microdialysis is a popular technique to measure neurotransmitters, including GLU, clinically,^5,10−14^ and in animal models,^15,16^ though it suffers from poor spatial (millimeter scale) and temporal (minute scale) resolution. Genetically encoded fluorescent reporters are powerful tools for real-time GLU detection.^17−19^ However, the technique requires genetic modification and an invasive imaging setup. In vivo electrochemical GLU sensors have the advantage of high temporal and spatial resolution, as well as more universal applicability regarding choice of location and organism to be studied.

Electrochemical GLU sensors based on glutamate oxidase (GluOx) have provided valuable data regarding in vivo GLU fluctuations in the central nervous system. These sensors utilize GluOx adhered to the electrode surface by a cross-linker (such as gluteraldehyde,^20,21^ chitosan,^22,23^ or polyethylene glycol diglycidyl ether^14,24^). GluOx reacts with GLU to produce α-ketoglutaric acid and hydrogen peroxide. The hydrogen peroxide is then oxidized at the electrode surface, and the resulting current is measured as a proxy for GLU concentration.^25^ To maintain selectivity, screening layers such as Nafion and m-phenylenediamine are deposited directly on the metal surface under the enzyme layer to provide charge or size exclusion.^26,27^ Sensors constructed in this manner have been used to study disease states including traumatic brain injury (TBI),^28,29^ cardiac ischemia,^30^ and Huntington’s disease.^31,32^ However, the major drawback of these sensors is the lack of stability over time. While sensors constructed with several methods have been demonstrated to function after days or weeks of incubation *in vitro* under various conditions,^22,33,34^ multiday GLU sensing with chronically implanted electrodes has only seen extremely limited use in vivo; currently, there are only examples of sensors lasting 7^35^ or 11^36^ days in vivo.

In this work, we explore the use of a platinum electrode surface with nanometer-scale roughness (nanoPt) to improve GLU sensors. Nanomaterials have previously been incorporated into various types of biosensors with great success in applications such as sensing in blood, cerebrospinal fluid, or food.^37−40^ Previously we have found that nanotopography improves the binding density and stability of biomolecules immobilized on electrode implants.^41−43^ Based on these findings, we hypothesized that the rough nanoPt surface results in an increased surface area to oxidize H_2_O_2_ and provides additional anchor points for GluOx to be bound to the surface, thereby increasing the sensitivity and stability of the GLU sensor. Additionally, increasing effective surface area without increasing the geometric area means that electrodes can have a smaller footprint without sacrificing signal intensity, allowing the fabrication of higher-density electrode arrays with a small cross-section.

We also investigated several potential failure modes of electrochemical GLU sensors by incubating sensors in several concentrations of H_2_O_2_ and GLU, compounds that are found in vivo and likely to cause the degradation of the enzyme sensors. In vivo, we show that nanoPt sensors outlasted smooth Pt sensors, which failed after only 3 days. We used the nanoPt sensor arrays to capture changes of GLU in rat striatum after a traumatic brain injury. We also demonstrated the ability to functionalize different microelectrodes of an implantable MEA and simultaneously measure GLU and γ-amino butyric acid (GABA) concentrations in porcine thoracic spinal cord, showing the capacity for multianalyte detection. Taken together, the nanoPt surface modification provides a simple method to improve the sensitivity and longevity of implantable microelectrode sensor arrays and has the potential to be expanded to other enzyme-based biosensors, including those for glucose^44−46^ and acetylcholine.^20,47^

## Methods

### Sensor Construction

Smooth Pt sensors were made using established methods.^48,49^ First, the electrode was immersed in a 10 mM solution of m-phenylenediamine (mPD, Sigma-Aldrich) in phosphate buffered saline (PBS), and 0.7 V was applied for 5 min to deposit the mPD film onto the Pt surface. mPD film serves as a screening layer to prevent electroactive species other than H_2_O_2_ from reaching the Pt electrode via size exclusion. Next, a solution containing 13.7 mg bovine serum albumin (BSA, Sigma-Aldrich) and 6.7 μL glutaraldehyde (Sigma-Aldrich) was prepared in 1 mL of DI water. BSA aids in cross-linking and has enzyme stabilizing effects.^50,51^ Nine μL of this solution was then added to 1 μL of a 1 U/μL GluOx solution (Cosmo Bio USA, Carlsbad, CA, USA) to make the final enzyme solution (final GluOx concentration of 0.1 U/μL). This was carefully dropped under a microscope onto GLU-sensitive electrode sites three times, with a short delay between drops to allow for drying. Sentinel control sites were constructed similarly, except GluOx was omitted from the solution. The GABA sensor used in the pig study was also constructed similarly to the GLU sensors, except with the addition of 0.1 U/μL of GABase (Sigma-Aldrich) to the final enzyme solution; the resulting sensors are sensitive to both GABA and GLU,^36,49,52,53^ as discussed in the Results and Discussion section. The MEA utilized for the dual GLU and GABA sensing was custom microfabricated as discussed below with electrode sites that are 200 μm apart to avoid crosstalk. Great care was taken to drop cast the appropriate enzyme solution only on the correct electrode site, with no spillover onto the electrode shank. MEAs with unacceptable coatings were discarded.

NanoPt sensors were constructed identically, except the nanoPt layer was deposited immediately prior to the mPD. Sensors were placed in a solution of 25 mM H_2_PtCl_6_ in 1 mM HCl (Sigma-Aldrich). Pt was reduced onto the surface by holding the electrodes at −0.3 V vs Ag/AgCl for 3 min using an Autolab potentiostat/galvanostat (PGSTAT128N, Metrohm, Herisau, Switzerland). NanoPt deposition was confirmed by a drop in impedance after deposition (see Figure S2). Optimization of deposition time revealed increasing deposition time in most cases produces diminishing returns in terms of increasing surface roughness. With both wire electrodes and our microfabricated MEAs, once the surface is sufficiently roughened, stacking rough platinum on top of rough platinum does not further decrease impedance.

For in vitro incubation experiment, 2 mm diameter Pt disk electrodes were used (CHInstruments, Austin, TX, USA). For the calibration curve and selectivity studies, the electrodes consisted of a 1 mm length of exposed Pt wire (0.005” diameter, Goodfellow, Huntingdon, UK). For the in vitro crosstalk study and in vivo mouse study, a commercial MEA was used (CM-style (mouse) or A style (crosstalk) single shank 16-channel 703 μm^2^ sites, 100 and 50 μm pitch respectively (NeuroNexus, Ann Arbor, MI, USA). For the in vitro sensitivity and selectivity tests and in vivo rat and pig study, in-house fabricated flexible MEAs were used. The details of the fabrication are discussed in the following section.

The in-house fabricated flexible MEAs were interfaced with the potentiostat with a custom printed circuit board (PCB). The PCBs were mounted with a zero insertion force (ZIF) connector to connect to the MEA and an Omnetics connector to interface with the potentiostat. A 16-channel passive headstage (Model RA16AC, Tucker-Davis Technologies, Alachua, FL, USA) was used to connect the Omnetics connector of the custom PCB or the NeuroNexus MEA to an in-house built breakout box. The breakout box interfaced with the potentiostat via alligator clips for electrochemical measurements.

### Flexible MEA Fabrication

A schematic representation of the steps of the flexible MEA fabrication is shown in Figure S1. The flexible MEAs have two layers of metal sandwiched in between three layers of polyimide. The MEAs were fabricated on a 4-in. silicon wafer with a 500 nm thick SiO_2_ layer (University Wafers Inc., USA). The wafer was first cleaned with acetone, isopropanol, and deionized (DI) water, sequentially, then dried and heated on a hot plate at 150 °C for 5 min and treated with O_2_ plasma using a reactive ion etcher (RIE, Trion Phantom III LT, Trion Technology, USA) for 1 min at 200 mTorr and 150 W. The cleaned wafer was then spin-coated with polyimide (PI) precursor HD-4100 (HD Microsystems LLC, Parlin, NJ, USA) at 3000 rpm for 1 min and soft baked at 90 °C for 3 min. The polyimide-coated wafer was then flood exposed with a mask aligner (Neutronix Quintel, NXQ400, Morgan Hill, CA) and cured in a tube furnace in a nitrogen (N_2_) environment at 350 °C for 1 h. To pattern the PI layer, positive photoresist AZ P4620 (MicroChemicals GmbH, Germany) was utilized as a mask. AZ P4620 was spun at 2000 rpm and baked at 105 °C for 5 min and photopatterned in the shape of MEA outlines with a maskless aligner (MLA, Heidelberg MLA100, Baden-Württemberg, Germany) and developed in AZ400 K 1:4 developer (MicroChemicals GmbH, Germany). The MEA outline was etched in RIE with a gas mixture of 4% SF_6_ in O_2_ plasma.

Next, the first metal layer was patterned via lift-off lithography. The surface was the PI layer on the wafer was treated with O_2_ plasma described as above and AZ P4210 photoresist (MicroChemicals GmbH, Germany) was spun at 3000 rpm and baked at 105 °C for 5 min. The photoresist was exposed using MLA and developed in AZ400 K developer 1:4 solution. The patterned area was cleaned with mild O_2_ plasma at 600 mTorr, and 60 W. A stack of metal 15 nm titanium (Ti)–100 nm gold (Au)–20 nm platinum (Pt) was evaporated on the patterned wafer in an electron beam evaporator (Plassys MEB550S, Angstrom Engineering, USA). The metal was lifted-off in acetone to define the electrodes, metal traces, and connection pads.

A second layer of PI was spun and cured as described above, and AZ P4620 was used as a mask to etch the second PI layer on top of electrode sites and connection pads. The PI layer was plasma activated and a second Ti–Au–Pt stack was deposited and patterned as described above forming a second layer of electrodes. Two metal layers were utilized to decrease the shank width while maintaining a high channel count. The final layer of PI was spun, cured, and etched following the procedures above but using a different pattern of AZ P4620 mask to form MEA outline and openings for electrodes. Lastly, the flexible MEAs were released from the silicon wafer by etching the SiO_2_ in buffered oxide etch (BOE 7:1, Transene, Danvers, MA). The final MEA dimensions are 5 mm shank length, 200 μm width, with 10 μm thickness. Electrode sites are 35 μm in diameter, spaced 200 μm apart, center to center.

### NanoPt Characterization and Data Analysis

Scanning electron microscopy (SEM) was used to characterize nanoPt deposition, set at 3.0 kV on a JSM 6335F SEM (Jeol USA, Peabody, MA, USA). Impedance measurements were taken from 10^5^ to 0.1 Hz with a sine wave oscillation and a 0.01 V RMS voltage using an Autolab potentiostat/galvanostat (PGSTAT128N, Metrohm, Herisau, Switzerland). All data was analyzed with MATLAB (Mathworks, Inc., Natick, MA, USA) and GraphPad Prism (GraphPad Software, San Diego, CA, USA).

Electrochemical active surface area (ECSA) was calculated by measuring the capacitance of the electrode and dividing by the specific capacitance of the electrode, estimated to be 20 μF/cm^2^ for a metal electrode in an electrolyte solution. Capacitance was measured by scanning CVs on an Autolab potentiostat/galvanostat between −0.2 and 1 V vs Ag/AgCl at different scan rates and plotting the current in the capacitive region (0.2 V) vs the square root of the scan rate and calculating the slope of the line of best fit.

### In Vitro Testing

For long-term in vitro stability testing, GLU sensors were made from platinum wire. Our control group was unmodified platinum, and the experimental group was platinum wire with nanoPt deposited. Then, the GluOx solution was drop-casted onto each sensor. Four incubation groups were used to test sensitivity stability: PBS, 100 μM sodium glutamate, 100 μM (low) H_2_O_2_, and 10 mM (high) H_2_O_2_ (*n* = 3 per incubation group each for bare and nanoPt). GLU sensors were incubated in these conditions for up to 21 days. To calibrate, each sensor was calibrated with 10 μM, 100 μM, and 1000 μM GLU standards. Amperometry was performed at 0.7 V vs Ag/AgCl with a CHInstruments 1430 potentiostat (CHInstruments, Austin, TX, USA). Current readouts were averaged across all sensors in each group for each group’s sensitivity curves over 21 days.

### In Vivo Surgical Procedures

All procedures involving animals were approved by the Institutional Animal Care and Use Committee of the University of Pittsburgh.

#### Chronic Glutamate Sensing in Mouse Striatum

**Figure 1:**
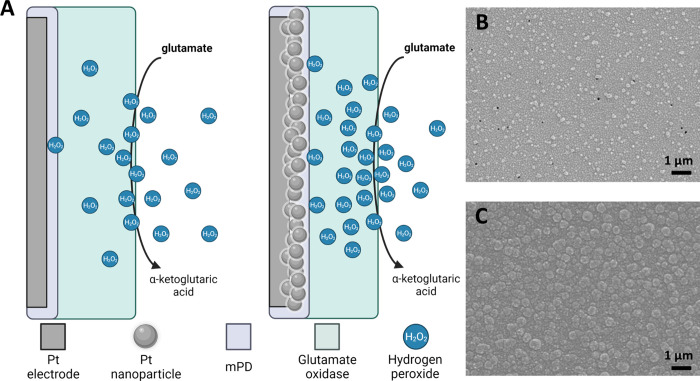
(A) Schematic representation of the proposed mechanism of the nanoPt GLU sensor. (B) Compared to smooth Pt, (C) NanoPt has a roughened surface.

To investigate the chronic performance of nanoPt sensors in vivo, a NeuroNexus CM-style probe was coated with alternating control (lacking GluOx) and nanoPt GLU sensor sites as described above (see Figure 1D). Of the 16 total sites, 6 were control GLU sensors, 2 were control sentinels, 6 were nanoPt GLU sensors, and 2 were nano Pt sentinels. A male mouse (C57BL/6J, 8–12 weeks, 22–35 g; Jackson Laboratory, Bar Harbor, ME, USA) was anesthetized, and the NeuroNexus probe was implanted under sterile conditions. The craniotomy was sealed with Kwik-Sil (World Precision Instruments, Sarasota, FL), and the probe was cemented in place with UV curing cement (Henry Schein, Melville, NY, USA). A 5 min amperometric measurement at 0.7 V vs Ag/AgCl was taken at each site using a CHI 1430 multipotentiostat. The mouse was allowed to wake up and return to its home cage. Every other day, the mouse was reanesthetized, a Ag/AgCl reference electrode was inserted subcutaneously; our previous work has shown that a replaceable subcutaneous reference results in better reference stability and thus more accurate applied potentials and avoids the tissue damage associated with a chronically implanted reference in the brain.^54^ Every other day, 5 min amperometric measurements were taken at each site and continued until all electrodes no longer had a measurable signal above the sentinel sites.

#### Striatal Glutamate Measurement during TBI in Rat

A male Sprague–Dawley rat (approximately 300 g, Charles-Rivers Inc., Wilmington, MA, USA) was anesthetized with isoflurane (5% for induction, 2.5% for maintenance) and placed into a stereotaxic frame (Kopf Instruments, Tujunga, CA, USA). The skin was resected, and the connective tissue was removed from the skull. A Leica ImpactOne (Leica Biosystems, Buffalo Grove, IL, USA) was used to create a controlled cortical impact (CCI) model TBI. A 5 mm diameter craniotomy was drilled posterior to bregma on the right hemisphere to allow the impactor to strike the cortex. A small burr hole was drilled over the striatum (AP 1.0 mm anterior to bregma, ML 2.5 mm lateral) on the ipsilateral side, and a flexible MEA (see fabrication details above) was inserted to a depth of 5.0 mm using a 50 μm diameter tungsten wire shuttle sharpened to a fine tip. Once at the final coordinates, the shuttle was carefully removed. Two additional burr holes were made in the contralateral hemisphere for the insertion of an AgCl-coated Ag wire reference electrode (Goodfellow, Huntingdon, UK) and a bone screw counter electrode. After MEA implantation, baseline GLU recording was performed with a CHI 1430 multipotentiostat (CHInstruments, Austin, TX, USA) at 3 channels simultaneously. While GLU sensing continued, the exposed dura was struck by the 5 mm impactor piston at a velocity of 4.00 m/s, with a 100 ms dwell time to a depth of 2.4 mm. This corresponds to a moderate TBI known to affect neurochemicals in tissue near the impact site.^45,55−57^ The impactor piston was angled 15° to accommodate the bulk of the piston as well as the stereotaxic micromanipulators holding it and the flexible MEA in place. After the conclusion of the experiment, the MEA was removed from the spinal cord and postcalibrated to confirm it remained functional with a 5-point calibration curve ranging from 10 μM to 1 mM.

#### Simultaneous Thoracic Spinal Glutamate and GABA Measurement in Pig

To test our sensors in vivo, we applied our nanoPt deposition to sense spinal cord GLU and GABA in the Yorkshire pig. The sensor testing experiment was performed after another cardiac study in which the effect of myocardial ischemia on spinal neural processing was assessed.^30^ The pig was initially anesthetized with isoflurane to perform invasive surgeries including T1-T4 levels laminectomy and sternotomy and then we used α-chloralose (50 mg/kg initial bolus followed by 20 mg/kg/h continuous infusion) to maintain anesthesia during the sensor testing. α-Chloralose was used to minimize the effects of anesthesia on the activity of spinal neurons.^58^ We adjusted the anesthesia level if needed by assessing corneal reflex, jaw tone, and hemodynamic indexes including heart rate and blood pressure. A water heating pad (T/PUMP; Gaymar Industries, Orchard Park, NY) was used to maintain the pig’s body temperature. Our flexible polyimide nanoPt MEA sensor was inserted into the dorsal horn of the T2-T3 spinal region using a micromanipulator. Throughout the acute experiment, amperometry was measured at 0.7 V vs Ag/AgCl with the CHInstruments 1430 multichannel potentiostat. GABA (10 mM) and α-ketoglutaric acid in PBS were injected into the spinal cord through an intrathecal catheter and spinal glutamate and GABA levels were measured. After the conclusion of the experiment, the MEA was removed from the spinal cord and postcalibrated to confirm it remained functional with a 5-point calibration curve ranging from 10 μM to 1 mM. The pig experiments were performed in compliance with the National Institutes of Health’s Guide for the Care and Use of Laboratory Animals.

## Results and Discussion

### NanoPt Increases Pt Surface Area and Improves Sensitivity

Previously, nanoPt coatings have been used to increase roughness and decrease microelectrode impedance for neural recording studies.^59,60^ Compared to a smooth Pt electrode (Figure 1B), the nanoPt surface is very rough (Figure 1C) with a cauliflower-like morphology. Because it is deposited electrochemically, individual sites on a single MEA can be selectively coated.

**Figure 2:**
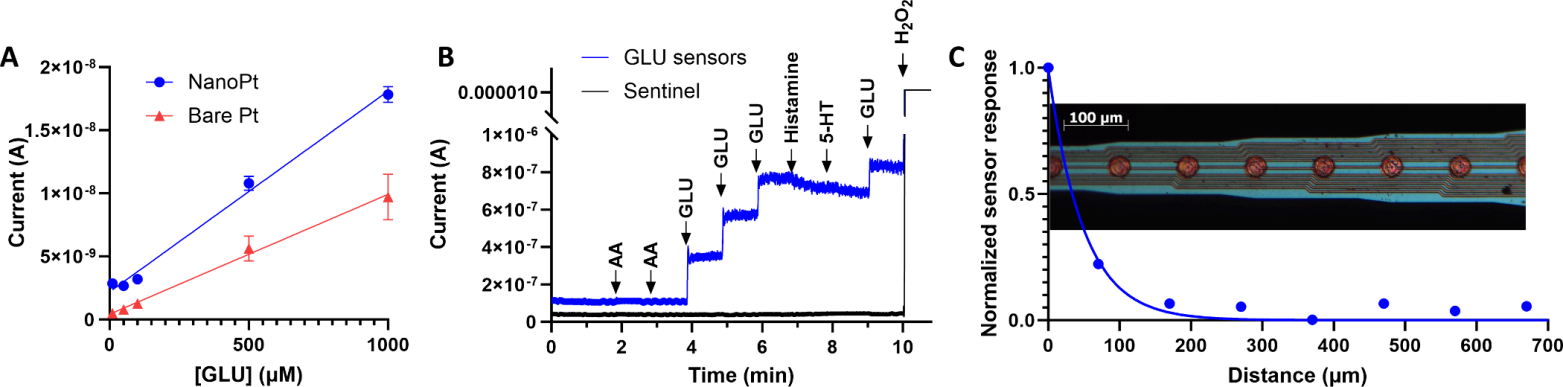
(A) NanoPt GLU sensors (blue) have significantly increased sensitivity to GLU compared to smooth Pt sensors (red). *n* = 3 in sensors (constructed from 125 μm diameter Pt wires) each group. Slope F-test, *p* < 0.0001. (B) GLU sensors constructed with a nanoPt layer (mean of *n* = 2, constructed from commercial Pt disk working electrodes, 2 mm diameter) are selective for GLU detection and do not respond to other analytes, except for H_2_O_2_. Sentinel control sites (*n* = 1) respond to H_2_O_2_ only. (C) To measure the diffusional crosstalk between sites, GluOx was drop cast onto only one site on a commercial MEA from Neuronexus. While the site closest to the GLU sensor site (70 μm away) detected some signal, further sites did not.

NanoPt significantly improves sensor sensitivity; when calibrated, nanoPt GLU sensors have 1.590 ± 0.057 × 10^–2^ nA/μM sensitivity, compared to the 9.50 ± 0.97 × 10^–3^ nA/μM sensitivity of a similarly constructed smooth Pt sensor lacking the nanoPt layer (Figure 2A). To confirm that nanoPt does not affect the selectivity provided by the mPD layer, nanoPt GLU and sentinel sensors were placed into a stirred 20 mL beaker of PBS, and 100 μL of 10 mM GLU, ascorbic acid (AA), serotonin (5-HT), and histamine were added. With the mPD size exclusion layer, the nanoPt maintains selectivity for GLU over the main interfering electroactive compound in the brain, ascorbic acid (AA), as well as other electroactive neurotransmitters like 5-HT and histamine (Figure 2B). The GLU sensor only responded to GLU and H_2_O_2_, and the sentinel control site only responded to H_2_O_2_. Interestingly, nanoPt also increases the off-rate constant (Figure S3). Taken together, the addition of a nanoPt layer to a GLU sensor is a facile and effective way of improving GLU sensor sensitivity without compromising selectivity.

We also sought to determine the effect of diffusional crosstalk on the detected currents from neighboring microelectrodes. Ideally, sentinel and sensor sites should be as close together as possible to ensure they are measuring the background response of the same tissue. However, the GluOx coating on a GLU sensor will produce H_2_O_2_ in the presence of GLU, which may evade oxidation, diffuse over to a sentinel site, and contribute to the sentinel current.^27,61^ The subtraction of an artificially high sentinel signal from the GLU signal will result in an artificially low calculated GLU concentration. Additionally, H_2_O_2_ oxidation can be confounded with pH in certain situations.^62^ Future attempts to detect multiple analytes on the same array must be free of interference from other analytes generated at adjacent electrode sites.

To investigate this, one site of a NeuroNexus A-style probe was coated with GluOx. We chose a commercial microfabricated MEA due to the precise spacing between electrode sites afforded by the manufacturing process and the stiff substrate. We then measured the GLU response at that sensor as well as the adjacent nonsensor sites to see how far the H_2_O_2_ from the GluOx would diffuse and still be detectable. The normalized data is shown in Figure 2C. The distances on the *X* axis are measured from the closest edge of the electrode to the edge of the coating spot, with *x* = 0 as the response of the actual sensor itself. The adjacent site had a response that was approximately 22% of the sensor, but the signal on the next site (approximately 170 μm from the sensor’s edge) decayed to 6%. Based on this finding, for our subsequent experiments using MEAs with mixed sensor types (i.e., GLU, sentinel, and GABA) we kept at least 170 μm between coating types to prevent diffusional crosstalk.

Sensors with roughened topography have been shown to have higher sensitivity compared to smooth counterparts.^63,64^ We calculated electrochemical active surface area and found an approximately 28-fold increase in the electrochemical active surface area (ECSA) after nanoPt deposition. *N* = 3127 μm diameter smooth Pt disk electrodes had an ECSA of 2.12 ± 0.45 × 10^–3^ cm^2^ surface area, which increased to 5.6 ± 2.0 × 10^–2^ cm^2^ after nanoPt deposition. Several coating types have been demonstrated to improve in vitro sensitivity and/or stability with Pt nanoparticles, including Pt nanoparticle-based ink,^65^ conducting polymer/Pt nanoparticle composites,^66^ and Pt nanoparticle-decorated multiwalled carbon nanotubes.^67^ We hypothesize that the nanoPt may be producing the observed sensitivity enhancement via three mechanisms, illustrated in Figure 1A: (1) the roughened surface will provide more anchor points for GluOx adherence,^41,42,68^ increasing the amount of enzyme per geometric area, thereby converting more GLU to H_2_O_2_. (2) The H_2_O_2_ generated by the GluOx is more likely to react with the Pt surface due to the increased surface area, resulting in a higher generated current. Finally, (3) oxidation and removal of more of the GLU and generated H_2_O_2_ may result in a more dramatic concentration gradient between the electrode surface and the bulk, resulting in increased GLU and H_2_O_2_ flux toward the electrode.^69^

### Investigating the Degradation Modes of GLU Sensors In Vitro

**Figure 3:**
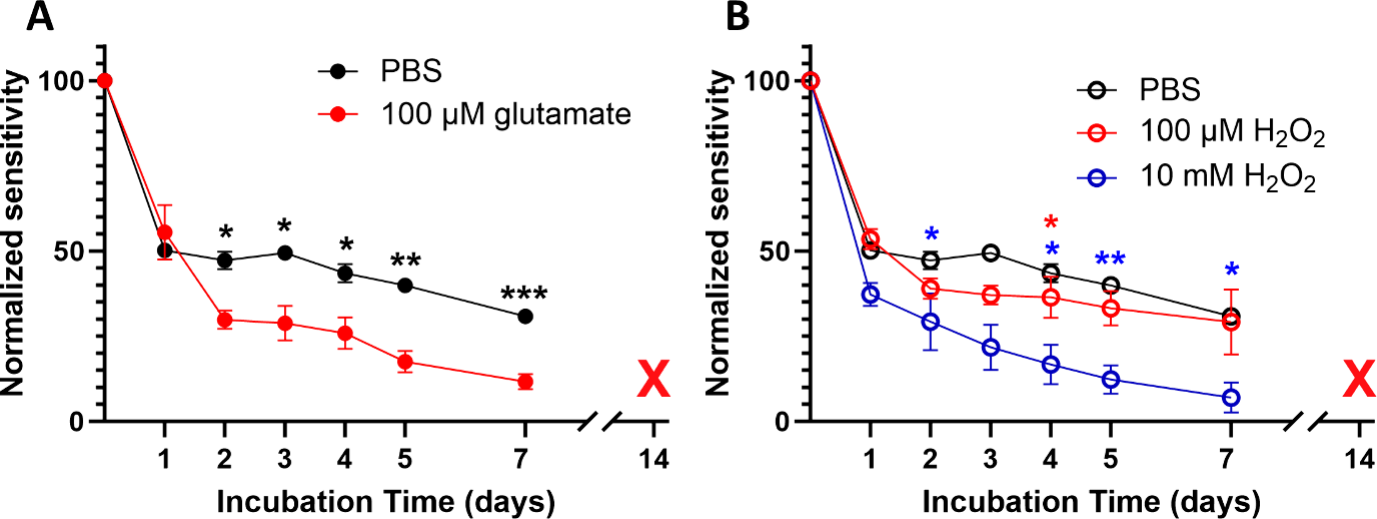
(A) Smooth Pt GLU sensors incubated in 100 μM GLU in PBS (red trace) have decreased sensitivity after 2 days of incubation, compared to sensors incubated in PBS only (black trace). (B) While 100 μM H_2_O_2_ (red) had no effect on sensitivity except on day 4, 10 mM H_2_O_2_ (blue) caused a significant decrease compared to PBS out to day 7. All sensors had failed when they were tested on day 14. The black PBS traces in both figures are identical and are presented in both figures for comparison. Two-way ANOVA with Dunnett’s multiple comparison test * = *p* < 0.05; ** = *p* < 0.005; *** = *p* < 0.0005.

While there are several reports of coatings and sensor construction methods that can improve GLU biosensor stability in vitro,^22,33,34^ there has only been a single report of chronic GLU sensing in vivo, to our knowledge.^35^ In vitro tests of enzyme biosensors typically involve incubation in 37 °C PBS. We decided to investigate two conditions that may influence sensor longevity in vivo, beyond temperature, pH, and salt concentrations. Sensors were made from wire due to the ease and low cost of manufacture so that several conditions could be tested. In vivo, the sensor is going to be exposed to GLU, and the GluOx bound to the surface is going to be reacting with it and producing H_2_O_2_, regardless of whether a measurement is actually being taken. To explore the effect of GLU on sensor longevity, smooth Pt wire GLU sensors (*n* = 3 in each group) were incubated in either PBS or 100 μM GLU (Figure 3A). 100 μM is a concentration similar to what we^30^ and others^70^ have previously reported in vivo. The exposure to GLU resulted in decreased sensitivity over time compared to the unexposed sensor, indicating that GLU sensor failure is accelerated by exposure to the enzyme’s substrate. This is in agreement with a previous study that indicated that high concentrations of glucose hastened the failure of sensors utilizing cross-linked glucose oxidase to generate H_2_O_2_.^71^

Another potential mechanism of GLU-induced failure could be the generation of H_2_O_2_. While H_2_O_2_ concentrations in the healthy brain are generally very low and well controlled by cellular peroxide disposal pathways (*t*_1/2_ = 2.2 s),^72,73^ at the surface of the sensor, they are significantly elevated due to the production of H_2_O_2_ as a byproduct of the conversion of GLU to α-ketoglutarate. Highly reactive H_2_O_2_ could potentially react with the GluOx and reduce its bioactivity. To determine the effect of H_2_O_2_ on sensor stability, we incubated sensors in either 100 μM or 10 mM H_2_O_2_ and found that while the 10 mM condition resulted in hastened sensor failure, the 100 μM condition did not, with the exception of day 4 (Figure 3B).

### NanoPt Improves Sensor Stability In Vitro

We hypothesize that the roughened surface provided by nanoPt will help improve the sensing stability of GLU sensors in two ways; first, the rougher surface presents more potential anchor points for cross-linking between GluOx and the electrode surface for more stable binding. Second, the increased surface area means that generated H_2_O_2_ is more likely to come into contact with the platinum electrode and be oxidized, allowing less to diffuse away and cause undesirable secondary reactions that may damage the electrode, coatings, or tissue.^63,64,69^

**Figure 4:**
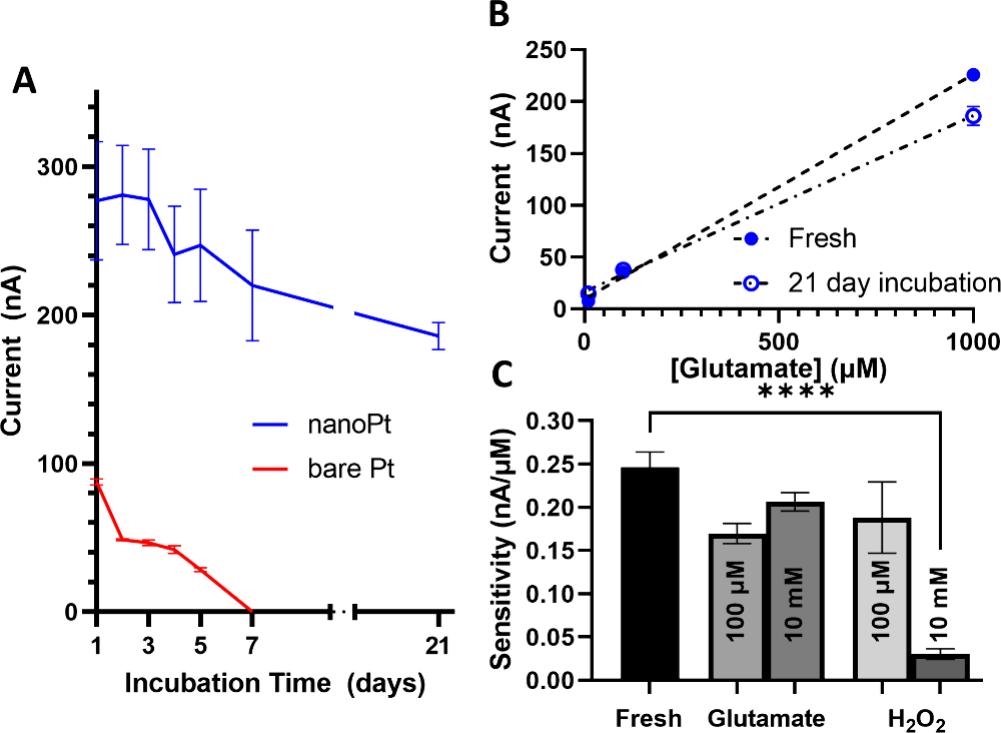
(A) After exposure to 100 μM GLU, smooth Pt sensors can no longer detect 1 mM GLU after 7 days (red). However, nanoPt sensors (blue) were still able to detect 1 mM GLU after 21 days of incubation. (B) The sensitivities of the nanoPt sensors after 21 days of incubation were only slightly decreased compared to the sensitivity immediately after they were prepared. (C) After 21 days of incubation, only incubation in 10 mM H_2_O_2_ resulted in a significant decrease in sensitivity compared to the initial sensitivity. One-way ANOVA, **** = *p* < 0.0005.

We explored the stability of nanoPt GLU sensors in vitro by subjecting them to the same tests as the smooth Pt sensors; namely, they were incubated for 21 days in either PBS, 100 μM GLU, 10 mM GLU, 100 μM H_2_O_2_, or 10 mM H_2_O_2_. We found that, in the most physiologically relevant condition—exposure to 100 μM GLU—the nanoPt sensors did not fail during the time window studied. Figure 4A shows the response of smooth Pt and nanoPt sensors to 1 mM GLU after incubation in 100 μM GLU (i.e., approximately the expected in vivo concentration). The response of the nanoPt after 21 days did not significantly decrease compared to day 1. On the other hand, the smooth Pt sensor did not have a detectable response after only 7 days. Figure 4B shows that there was no significant change in the calibration curves of the nanoPt sensors after they were freshly prepared and after 21 days of incubation. Indeed, the only condition that affected the sensitivity of the nanoPt sensors after 21 days was 10 mM H_2_O_2_, a supraphysiological condition that is unlikely to occur in vivo.

### NanoPt Improves Sensor Stability In Vivo

The development of robust chronic neurotransmitter sensing systems that can investigate the brain in the days and weeks following implantation is of great interest. For investigations correlating neurotransmitters to animal behavior, a postsurgical recovery period is required to ensure there are no confounding effects of the surgery or anesthesia on behavior or neurotransmission.^74,75^ Investigating the progression of disease, treatment, and recovery over time is also of interest.^36,56,76−80^ Generally, separate populations of animals are required for each time point, but advances in sensor stability may give researchers access to high-quality longitudinal neurotransmitter data within a single population of animals. While there are certainly many reports of enzyme biosensors with nanomaterial coatings that have improved in vitro stability, in vivo stability is only rarely investigated.^35,36^

**Figure 5:**
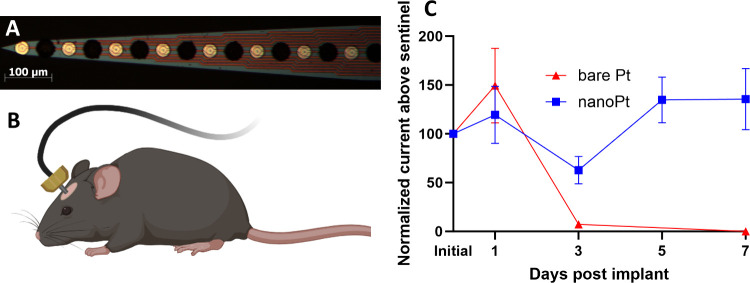
(A) Neural probe used to study in vivo GLU sensor stability with alternating sites coated with nanoPt (black sites). (B) Schematic of the GLU electrode stability experiment. The electrode from (A) was implanted chronically with measurements taken for a week after surgery. (C) Chronic glutamate sensing in a mouse. While nearly all smooth Pt sensors failed in vivo by day 3 post implantation, several GLU sensors were still detecting GLU after 7 days in vivo. By the next measurement (day 9), all sensors had failed.

While the nanoPt sensors demonstrated a dramatic increase in stability compared to smooth Pt sensors in vitro under several conditions, we sought to examine their stability in vivo. Electrode sites on a CM-style NeuroNexus probe were coated with either nanoPt or left bare in an alternating manner (Figure 5A). A commercial MEA was chosen for this experiment because it is well-established to maintain functionality in vivo for electrophysiology studies for many months,^42,81^ so we could be confident that any observed sensor failure is not a result of the failure of the underlying electrode. Of the eight nanoPt sites, six were functionalized into GLU sensors, and two as sentinels. Similarly, of the eight smooth sites, six were GLU, and two were sentinels. The probe was implanted into the striatum of a mouse, and GLU measurements were performed regularly until all electrodes had no quantifiable current above the sentinel sites (Figure 5B). Figure 5C shows that, while the smooth sites performed well for 24 h, by day 3 nearly every site had failed. In comparison, several of the nanoPt sites were still functional for 7 days postimplantation, though by day 9, all sites had failed.

The nanoPt sites demonstrated significantly improved stability compared to the smooth sites; however, they were only functional for 7 days compared to over 3 weeks in vitro. The only condition that resulted in a significant decrease in sensitivity in vitro was exposure to an extremely high concentration of H_2_O_2_—10 mM. This would indicate that our in vitro tests either did not capture the dominant failure mechanism in vivo or that the local concentration of H_2_O_2_ generated around the electrode sites is extremely high. Failure modes not tested in the in vitro experiments, including proteases and host immune response, are very likely contributors to loss of function in vivo. It is also possible that H_2_O_2_ could be causing additional inflammation beyond that usually elicited by a typical neural probe, increasing tissue damage and hastening electrode failure. The tissue response to GluOx-coated sensors will be studied in future work.

While in general we found that increasing deposition time usually does not result in further decreased impedance past a certain point, one interesting exception to this is the NeuroNexus probe used in this experiment. In this case, the electrodes on the array are flush with or raised above the silicon substrate. This allows nanoPt to build up along the edges, overgrow the electrode site, and further increase electrode surface area (see Figure 5A). While we did not optimize this specific case and used the same deposition time (3 min) as the rest of the experiments in this work, it is likely that increasing deposition time will increase functional surface area and further improve sensitivity on this type of electrode. Further optimization on specific styles of electrode is warranted, but potential drawbacks of longer deposition times include mechanical instability due to the weight of the extra metal and the potential for electrical shorts between adjacent electrode sites.

### Flexible MEA Enables GLU Sensing during Mechanical Injury

**Figure 6:**
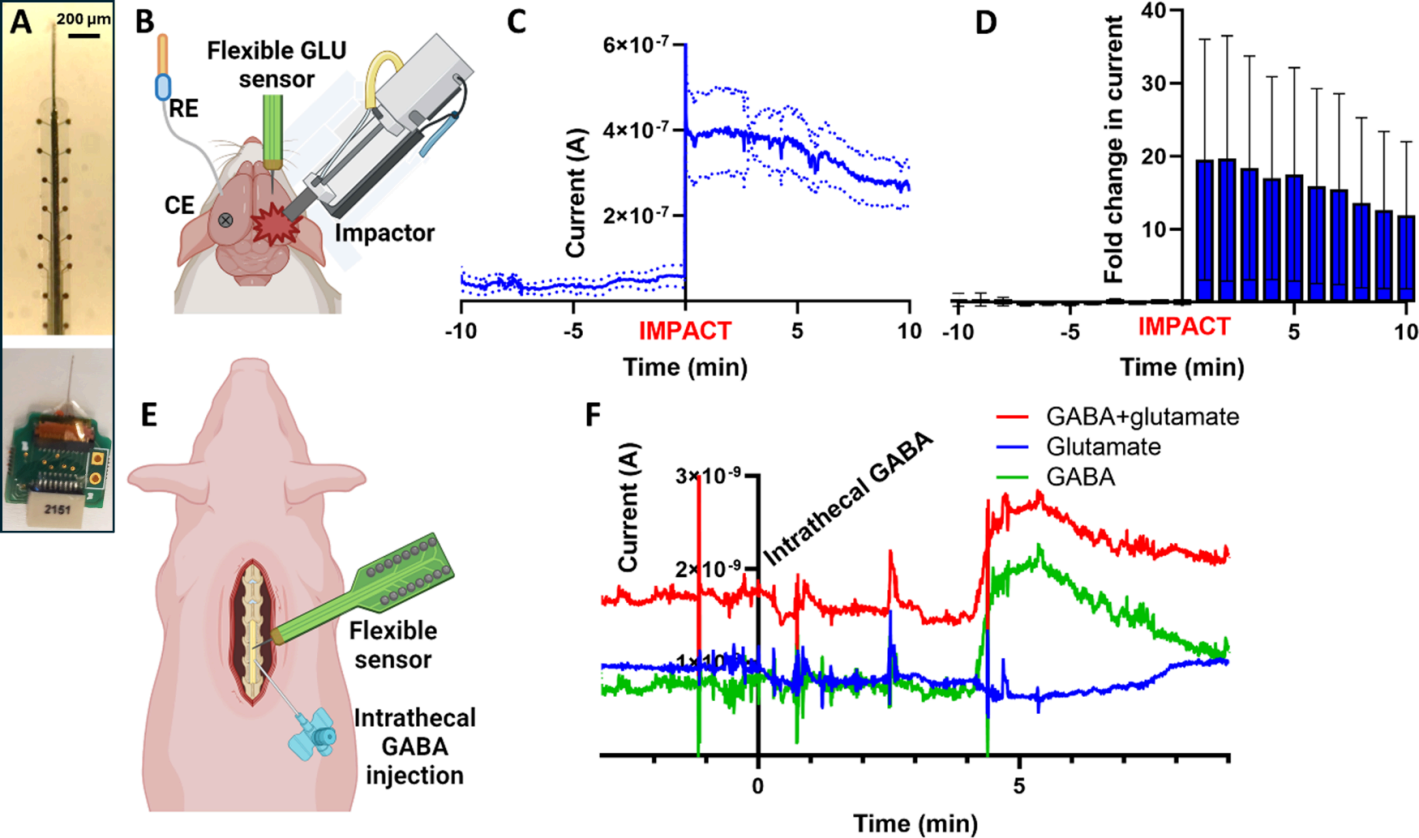
(A) A photo of an assembled flexible electrode array with a sharpened tungsten wire (top) and a photo of the MEA in the ZIF connector of the custom PCB (bottom). An Omnetics connector is used to interface with the potentiostat. (B) Schematic representation of the setup for recording GLU concentrations during TBI. (C) The stable GLU baseline rose dramatically after the cortical tissue was struck by the impactor. (*n* = 3 sites recorded simultaneously). (D) For 10 min post impact, GLU signals remained elevated over 10-fold above baseline (baseline GLU defined as the last data point collected before impact). (E) Schematic representation of the experimental procedure for measuring GLU and GABA in the porcine thoracic spinal cord. A flexible MEA functionalized for GLU and GABA sensing was inserted into the thoracic spinal cord. GABA was injected near the implantation site. (F) Simultaneous detection of GLU and GABA in the pig spinal cord. At *t* = 0 min, GABA is injected through a catheter into the spinal cord a few mm away from the sensor.

As a proof of concept, we used the nanoPt sensors on a flexible MEA to track GLU concentration changes induced by traumatic brain injury in rats. As shown in Figure 6B, a nanoPt GLU sensor constructed with an in-house fabricated flexible MEA (photo in Figure 6A) was inserted into the striatum of a rat with the aid of a sharpened tungsten guide wire. A reference electrode and a bone screw counter electrode were inserted into the contralateral hemisphere. A large craniotomy was performed posterior to bregma and the GLU sensor. After a 10 min baseline GLU measurement, the exposed dura was struck with an impactor. GLU concentrations rose immediately and remained elevated for the duration of the experiment (Figure 6C). Compared to the baseline, GLU current increased approximately 20-fold (Figure 6D). This is in agreement with other reported GLU concentrations before and after TBI.^29^ While the previous study measured the GLU with stiff MEA sensors in separate animals (sham and TBI) to deduce the difference in GLU, to our knowledge, the current study is the first report of GLU sensing throughout the cortical impact injury process from the same animal. This is made possible by the ultraflexibility of the thin film polymer substrate of the MEA that can accommodate the tissue displacement caused by the physical impact. Flexible GLU sensors have previously been used to reduce tissue inflammation due to brain micromotion,^33,82,83^ and we have previously shown that motion artifacts in sensing data are dramatically reduced in flexible probes compared to traditional stiff probes of similar dimensions.^30^

### Multianalyte Sensing in Large Animals

One of the advantages of MEA-based electrochemical sensors is their customizability toward specific applications. Multianalyte detection can be easily achieved by functionalizing different electrode sites for sensing different targets. Previously, we used smooth Pt sensors to investigate thoracic spinal GLU signaling during cardiac ischemia induced by left anterior descending artery (LAD) occlusion in pigs and found that cardiac ischemia causes sustained elevation in thoracic spinal GLU concentrations.^30^ In addition to GLU, GABA is an important inhibitory neurotransmitter involved in the pathology of many neurological diseases such as ischemic brain injury,^84^ Parkinson’s disease,^85^ and addiction.^86^ Evidence suggests that GABAergic neuromodulation is responsible for the reduction of cardiac arrhythmias observed with spinal cord stimulation during cardiac ischemia.^87,88^

In this study, we investigated if GABA and GLU can be sensed simultaneously in the pig spinal cord (Figure 6E). The microelectrode sites of a flexible nanoPt-coated MEA were functionalized for sensing either GLU, GABA, or as a sentinel control. The GABA sensor detects both GABA and GLU (Figure 6F, red), so the pure GABA (green) signal is calculated by removing the GLU contribution (blue) from the signal. Sites were spread across the electrode array in a pattern to ensure that GABA sensors were always near a GLU site, and GLU sites were always near a sentinel. The flexible MEA was custom microfabricated to have electrode sites that are 200 μm apart to avoid crosstalk between channels. When drop casting the enzyme coating, great care was taken to ensure that the coating did not extend past the electrode site onto the shank.

The MEA was implanted into the dorsal horn of the thoracic spinal cord of the pig to record simultaneous GLU and GABA signals. To help confirm that signals recorded at the GABA site were truly derived from GABA, 10 mM of GABA and α-ketoglutaric acid in PBS were injected intrathecally through a catheter a few mm away from the sensor (injection at *t* = 0 min). After approximately 4 min, the current increased at the GABA electrode, while simultaneously the current slightly decreased at the GLU electrode. We believe the increase in the GABA current to be a result of the direct detection of the injected GABA, with a time delay caused by the need for the GABA to diffuse from the injection site to the sensor. We hypothesize that the slight GLU current decrease is due to the inhibitory effect of GABA on the firing of glutamatergic neurons.

In this study, we utilized several different electrode designs for various applications: in vitro experiments were conducted with either commercial Pt electrodes or electrodes constructed from Pt wire, while in vivo experiments used commercial MEAs purchased from Neuronexus or in-house fabricated flexible MEAs. The expense of either commercial or fabricated MEAs may preclude technology dissemination, though small-diameter Pt wire has been used for in vivo sensing^70^ and may be a more cost-effective alternative. Carbon fiber microelectrodes have also been utilized as biosensors for analytes including glucose and lactate with cross-linked oxidase enzyme.^89^ nanoPt coated on carbon fiber may be a useful tool to simultaneously take advantage of the catalytic effect of Pt on the oxidation of H_2_O_2_ and the incredibly small size and cost-effectiveness of carbon fiber microelectrodes, though that remains to be explored.

## Conclusions

The addition of a nanoPt layer to Pt-GluOx GLU sensors is a simple, effective way to improve sensitivity and longevity without compromising selectivity. The additional surface area provided by the rough nanoPt provides additional anchor points to secure the GluOx to the surface, preventing enzyme loss over time. The additional surface area also oxidizes more of the produced H_2_O_2_. This provides a 2-fold benefit: more oxidized H_2_O_2_ results in a higher electrode sensitivity and prevents the H_2_O_2_ from undergoing undesirable reactions with the electrode, enzyme, or surrounding tissue. In vitro soaking tests in various stressor solutions revealed that the nanoPt GLU sensors were able to withstand high concentrations of GLU and H_2_O_2_ for at least 3 weeks without significant sensitivity loss, unlike GLU sensors prepared on smooth Pt electrodes. Only extreme H_2_O_2_ concentrations were able to destroy the nanoPt sensor in vitro. A stability test in vivo revealed that nanoPt GLU sensors could survive up to 7 days in vivo while smooth sites on the same array failed on day 3. The discrepancy with the longer in vitro stability for both sensor types reveals the importance of in vivo testing during sensor development.

We employed our nanoPt GLU sensor onto both commercial neural probes as well as in-house fabricated flexible MEAs. We detected in vivo GLU in rat brain immediately before, during, and after TBI using flexible nanoPt GLU sensors. We also measured fluctuations in GLU and GABA resulting from direct, intrathecal GABA injection in the porcine thoracic spinal cord. This demonstrates multianalyte sensing in large animal models, an important step for clinical translation, as well as other analytes using enzyme biosensors. A nanoPt layer is a simple way to build high-sensitivity, more robust biosensors harnessing enzymes to detect nonelectroactive analytes in vivo.

## Supporting Information Available

Fabrication schematic, EIS measurements, and flow injection analysis experiments (PDF)
