# Supplementary material for: Improving Sensitivity and Longevity of In Vivo Glutamate Sensors with Electrodeposited NanoPt
Source: ACS Appl Mater Interfaces. 2024 Jul 30;16(31):40570–80. doi: 10.1021/acsami.4c06692 (PMC11310907; doi:10.1021/acsami.4c06692)
Supplement: Supplementary file 1 — am4c06692_si_001.pdf [file am4c06692_si_001.pdf]

Supporting Information

Improving Sensitivity and Longevity of In Vivo Glutamate Sensors with Electrodeposited nanoPt

Elaine M. Robbins[1], Benjamin Wong[1,2], May Yoon Pwint[1,3], Siamak Salavatian[1,2], Aman Mahajan[1,2], Xinyan Tracy Cui[1, 3, 4, *]

[1]Department of Bioengineering, University of Pittsburgh, Pittsburgh, PA, USA

[2]Department of Anesthesiology & Perioperative Medicine, University of Pittsburgh School of Medicine, Pittsburgh, PA, USA

[3]Center for Neural Basis of Cognition, University of Pittsburgh, Pittsburgh, PA, USA

[4]McGowan Institute for Regenerative Medicine, University of Pittsburgh, Pittsburgh, PA, USA

*Corresponding author. Department of Bioengineering, University of Pittsburgh, 5057 Biomedical Science Tower 3, 3501 Fifth Avenue, Pittsburgh, PA, 15260, USA. E-mail address: xic11@pitt.edu

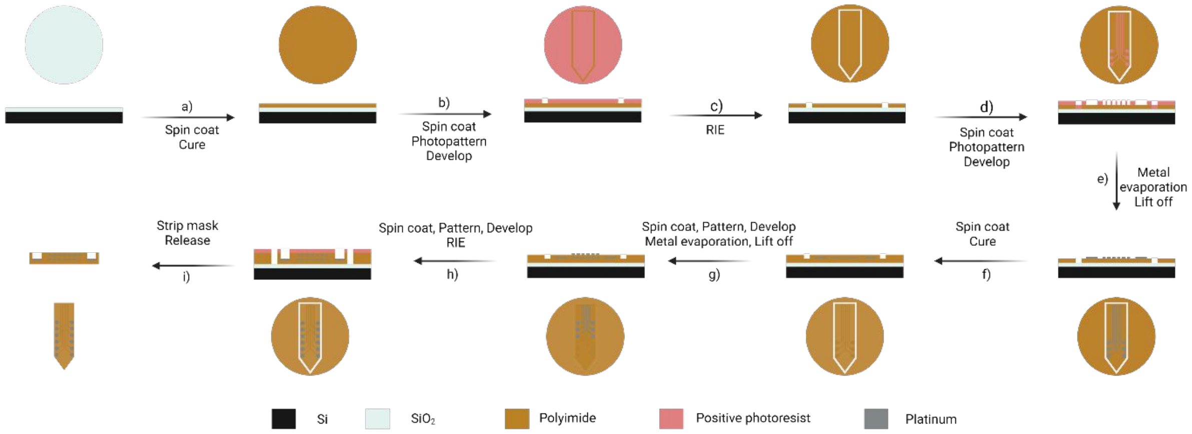

Figure S1: Schematic representation of the fabrication steps for the flexible MEAs used in the pig and rat in vivo experiments.

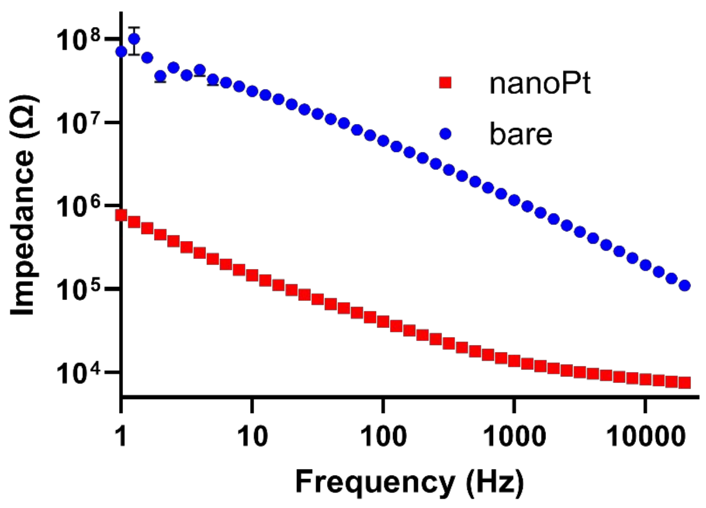

Figure S2: EIS of 13 sites of a Neuronexus probe before (blue) and after (red) nanoPt coating.

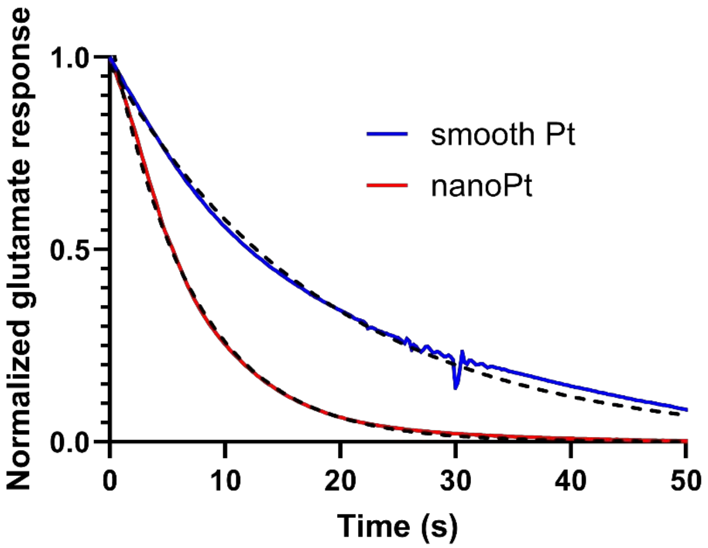

Figure S3: Off rate times for smooth Pt (blue) vs nanoPt (red). After exposure to a high concentration of glutamate in a flow cell, smooth Pt and nanoPt glutamate sensors were allowed to return to baseline. The data was normalized and fit to the exponential decay function $f(t) = \exp(-t/\tau)$, where τ is the mean lifetime. The 95% confidence interval for τ was 18.3-18.8 s for smooth Pt and 6.6-8.5 s for nanoPt.
